# Optical Trapping and Manipulation of Superparamagnetic Beads Using Annular-Shaped Beams

**DOI:** 10.3390/mps1040044

**Published:** 2018-11-20

**Authors:** Leandro Oliveira, Warlley H. Campos, Marcio S. Rocha

**Affiliations:** Laboratório de Física Biológica, Departamento de Física, Universidade Federal de Viçosa, Viçosa 36570-900, Brazil; leandro.o.oliveira@ufv.br (L.O.); warlley.campos@ufv.br (W.H.C.)

**Keywords:** optical tweezers, optical trapping, superparamagnetic particles, trap stiffness, annular beam

## Abstract

We propose an optical tweezers setup based on an annular-shaped laser beam that is efficient to trap 2.8 μm-diameter superparamagnetic particles. The optical trapping of such particles was fully characterized, and a direct absolute comparison with a geometrical optics model was performed. With this comparison, we were able to show that light absorption by the superparamagnetic particles is negligible for our annular beam tweezers, differing from the case of conventional Gaussian beam tweezers, in which laser absorption by the beads makes stable trapping difficult. In addition, the trap stiffness of the annular beam tweezers increases with the laser power and with the bead distance from the coverslip surface. While this first result is expected and similar to that achieved for conventional Gaussian tweezers, which use ordinary dielectric beads, the second result is quite surprising and different from the ordinary case, suggesting that spherical aberration is much less important in our annular beam geometry. The results obtained here provide new insights into the development of hybrid optomagnetic tweezers, which can apply simultaneously optical and magnetic forces on the same particles.

## 1. Introduction

The optical and magnetic manipulation of microparticles has become in the past few years indispensable tools in biophysics and soft matter research [1,2,3,4]. In fact, fields such as single-molecule biophysics [5,6], cell biology [7,8] and colloidal science [9] have experienced considerable advances with the advent of the tweezers techniques, namely the optical and magnetic tweezers. These techniques allow one to apply forces and torques on the particles of interest, which are usually attached to the systems that one intends to manipulate, stretch and/or rotate.

Each of these techniques has its own specific advantages. Optical tweezers, for instance, allow direct local force application with high spatial and temporal resolutions [3,10]. Magnetic tweezers, on the other hand, can be conveniently used as a force clamp and to apply torque on the systems of interest [10,11,12]. An apparatus that allows one to explore the best features of the two tweezers techniques would therefore be of great interest.

Here, we propose and characterize an optical tweezers setup based on an annular-shaped laser beam that is efficient to trap 2.8 μm-diameter superparamagnetic particles, thus allowing the development of a hybrid instrument—optomagnetic tweezers—which allows one to apply simultaneously optical and magnetic forces on the same particles. The idea is to trap the superparamagnetic particles optically, which can be naturally manipulated with permanent or electromagnets. These particles usually are more difficult to trap stably by single Gaussian beam optical tweezers because they considerably absorb light, which generates relevant radiation pressure and radiometric forces on the particles, pushing them away from the focal region [13,14]. We have circumvented this problem here by changing the laser intensity profile, using a phase contrast mask to produce an annular-shaped beam. This approach has been proven very efficient to trap our 2.8 μm-diameter superparamagnetic particles optically, resulting in stable optical tweezers with some peculiar features that we have fully characterized in the present work.

Some instruments and/or approaches that take advantage of applying both optical and magnetic forces simultaneously on particles have been previously reported in the literature [13,14,15,16,17,18,19,20,21]. Nevertheless, most approaches use Gaussian beam optical tweezers to manipulate the magnetic beads, which can result in non-negligible heating of these beads (and consequently, of the surrounding medium) due to laser absorption, resulting in an unstable optical trap depending on the laser power and/or bead size [14]. Differently, our approach strongly reduces the laser absorption by cutting off the middle of the original Gaussian beam, which is the beam portion that mostly contributes to the radiation pressure and radiometric forces on the particles, i.e., the forces that disturb the stable trapping of such particles [22,23]. The present work focuses on the characterization of the optical trapping of superparamagnetic beads with the annular beam tweezers. Although it is well known that such beads can also be manipulated using a magnetic field in magnetic tweezers, thus allowing the development of a hybrid instrument, here, we do not present any measurement of the magnetic forces on such beads.

## 2. Materials and Methods

**Figure 1:**
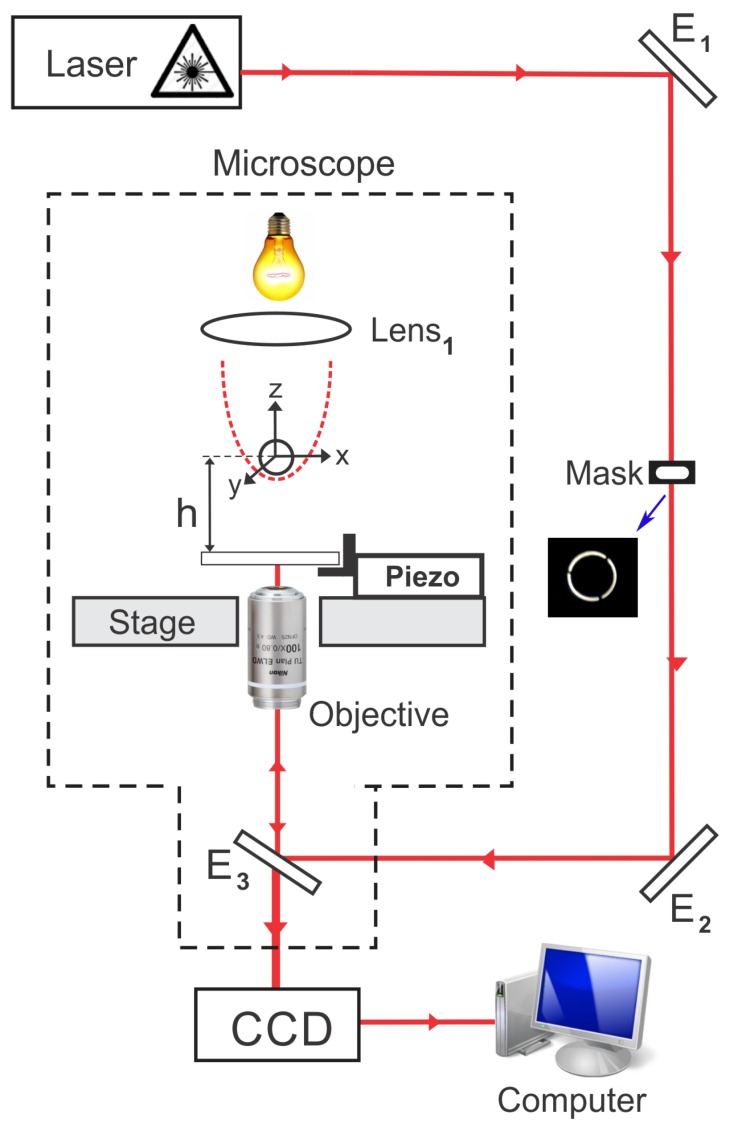
Schematic drawing of the experimental setup, with a photograph of the phase mask used. The mask is positioned at the optical path outside the microscope, as shown in the figure. Basically, the tweezers are mounted with a 1064-nm laser in an inverted microscope. A charge-coupled device (CCD) camera is used to visualize the beads and to record the experiments. $E_{1}$, $E_{2}$ and $E_{3}$ are mirrors. *h* is the distance from the bead center to the coverslip surface.

Our tweezers are mounted in a Nikon (Tokyo, Japan) Ti-S inverted microscope using a 100× NA 1.4 oil immersion objective with an entrance aperture radius of (3.50 ± 0.05) mm. The laser is a IPG Photonics (Oxford, MT, USA) Model YLR-5-1064-LP with λ = 1064 nm and a maximum output power of 5 W, with linear polarization. The laser is originally in TEM${}_{00}$ mode, with a Gaussian intensity profile, which is the most used configuration for optical tweezers. The Gaussian beam is converted into an annular beam by using a phase contrast mask (Nikon Ph1, with an inner and outer radius of 2.9 mm and 3.6 mm, respectively), which produces the required intensity profile. A similar profile can also be easily implemented in any optical tweezers setup using a spatial light modulator. Figure 1 shows a schematic drawing of this setup, with a photograph of the phase mask used.

The superparamagnetic beads used were the M-280 Dynabeads (Carlsbad, CA, USA), which have a 2.8 μm diameter (Invitrogen Cat. 65801D). They were diluted in deionized water before use. According to the manufacturer, the M-280 beads are composed of a polystyrene matrix with an even dispersion of magnetic material (Fe${}_{2}$O${}_{3}$ + Fe${}_{3}$O${}_{4}$) throughout the beads. The iron content (Fe) corresponds to 12% by weight for the beads used. The sample chamber consists of an O-ring glued on a coverslip surface, where the working solution (deionized water + beads) is deposited to perform the experiments.

To characterize the optical trapping of such beads, we have performed systematic measurements of the tweezers’ trap stiffness κ as a function of the laser power and also as a function of the bead height (distance from the bead center to the coverslip surface). The measurements of the trap stiffness κ were performed by using two independent methods: the drag force and the equipartition methods, both described in [24]. In order to compare the measured trap stiffness with a theoretical prediction of the optical forces, we have performed calculations in the geometrical optics (GO) regime [25]. These calculations were carried numerically using the procedures described in detail in [26].

## 3. Results and Discussion

Before performing the force measurements and calculations, the relevant optical parameters of our system were carefully measured. The knowledge of such parameters is fundamental to characterize the trap that was used to perform the experiments. In addition, they are essential to perform the numerical calculations of the optical forces and consequently to obtain the theoretical prediction of the trap stiffness κ that we compare with our experiments.

### 3.1. Characterization of the Laser Intensity Profile

**Figure 2:**
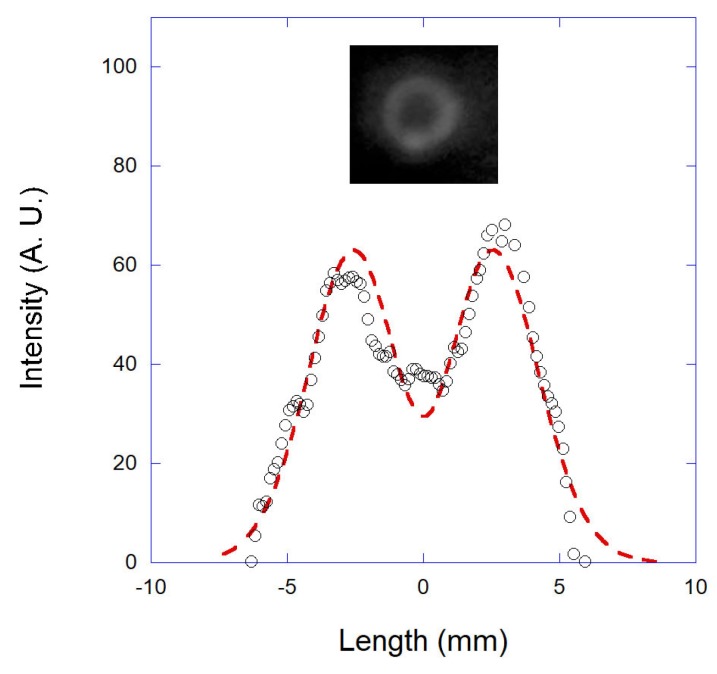
A photograph of the annular-shaped beam at the objective entrance, with the intensity profile measured along its diameter (black circles). The dashed red line is a fitting to the proposed model for the intensity profile of the annular beam, Equation (1).

In Figure 2, we show the annular beam photographed at the objective entrance and the respective intensity profile measured along its diameter (black circles). The dashed line shown in the figure is a fitting to the following proposed intensity profile for the annular beam:

(1) $$I=I_{1}\exp\left[\frac{-2{\left(\rho -\rho_{0}\right)}^{2}}{\sigma_{1}^{2}}\right]-I_{2}\exp\left[\frac{-2{\left(\rho -\rho_{0}\right)}^{2}}{\sigma_{2}^{2}}\right].$$

Equation (1) is the subtraction of two simple Gaussian beams with different waists ($\sigma_{1}$, $\sigma_{2}$) and intensities ($I_{1}$, $I_{2}$), centered at the same position $\rho_{0}$. This is a very simple model capable of fitting the measured intensity profile with accuracy, and the relevant parameters can be determined from the fitting with very small error bars: $\sigma_{1}$ = (4.5 ± 0.1) mm, $\sigma_{2}$ = (3.6 ± 0.1) mm and $I_{2}$ = (0.91 ± 0.01)$I_{1}$ (note that these intensities are measured in arbitrary units). In principle, other functions could be used to describe the measured intensity profile. However, the chosen function has the advantage of being mathematically simple, thus simplifying the numerical calculations of the optical forces.

### 3.2. Characterization of the Objective Transmittance

The characterization of the objective transmittance is important to allow one to know the local laser power reaching the trapped beads. Generally in optical tweezers experiments, people usually measure the laser power at the objective entrance, because such a measurement can be easily done with ordinary power meters based on photodetectors. Such power meters usually accurately measure only parallel light beams, underestimating the power of highly focused light beams. Thus, they cannot be simply positioned beyond the objective lens to measure the laser power that reaches the sample. Therefore, one must measure the objective transmittance to know the actual laser power that reaches the beads in order to perform the theoretical prediction of the optical forces and trap stiffness.

The objective transmittance depends on the characteristics of the objective lens itself (lens type, numerical aperture, etc.) and of the laser used (wavelength, waist, intensity profile, etc.) [27]. Some different methods were developed in the past few years to allow the measurement of such parameter [27,28]. Nevertheless, recently, some power meters have emerged on the market that can be used to measure the power of focused beams with high accuracy. Here, we employ one of these power meters (ThorLabs Slide Power Sensor S170C, Newton, NJ, USA ) to measure the laser power directly at the objective focus for our focused annular beam. Thus the objective transmittance was calculated by dividing such power by the measured power at the objective entrance. We obtained $T_{annular}$ = (9 ± 1)%. For comparison purposes, the transmittance for the original Gaussian beam (without the phase mask in the optical path) was determined with the same procedure, with $T_{Gaussian}$ = (31 ± 1)%. This result for the transmittance of the Gaussian beam agrees with the results previously obtained with other approaches [27,28].

One should note that the transmittance obtained for the annular beam is considerably smaller than the one obtained for the original Gaussian beam. This result reflects the fact that the paraxial rays are transmitted more than the more angled rays by the objective lens. In fact, in [27], it was experimentally demonstrated that the transmittance of the objective used in the present work is a Gaussian function of the radial distance, i.e., it is maximum for paraxial rays and decays for edge rays. It also implies that the annular beam tweezers typically exert smaller forces on the beads compared to the original Gaussian beam tweezers for a fixed given laser power at the objective entrance. This may be a disadvantage for dielectric particles, but the annular intensity profile is essential to trap our superparamagnetic particles, which could not be stably trapped with our original Gaussian beam. Such result suggests that, depending on the situation, and especially when radiation pressure and heating effects are non-negligible, annular-shaped beams are more efficient to trap objects.

### 3.3. Numerical Calculation of the Optical Forces and Trap Stiffness in the Geometrical Optics Regime

As mentioned before, all the numerical results were obtained using the calculations fully described in [26], performed in the GO regime. We have used Equation (1) to describe the intensity profile of the beam and the transmittance $T_{annular}$ = (9 ± 1)% to calculate the local laser power on the superparamagnetic beads. We have adopted the value of 1.6 as the refractive index for the beads, since they are composed mainly of polystyrene, as cited in Section 2. Results obtained by other authors corroborate this assumption [15]. All the calculations reported here refer to the transverse force exerted by the tweezers on the beads, i.e., the component of the optical force parallel to the coverslip surface (plane xy). The optical axis of the system is then adopted as the *z* axis, as depicted in Figure 1.

**Figure 3:**
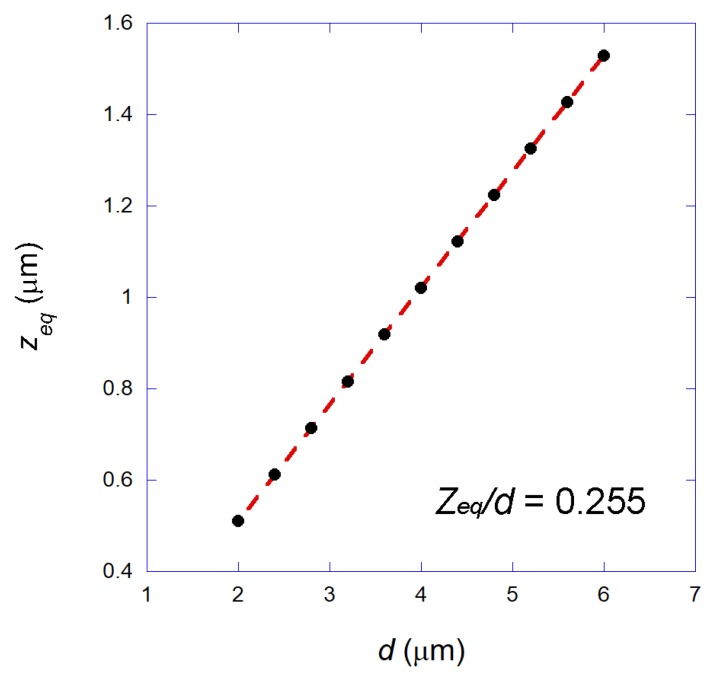
Black circles: Calculated equilibrium position of the trapped bead $z_{eq}$ as a function of the bead diameter *d*. Dashed line: A linear fitting. The geometrical optics (GO) calculations for our annular beam found a constant ratio $z_{eq}$/*d* = 0.255.

Firstly, in order to calculate the forces that a bead experiences close to the focal region, it is necessary to calculate the equilibrium position of the bead relative to the focus, since the bead center does not coincide exactly with the geometrical focus position due to the radiation pressure force [24,26]. Our GO calculations show that the bead stays in equilibrium at the optical axis at a certain position $z_{eq}$ above the focus. For a fixed bead size, the equilibrium position is independent of the laser power. For our 2.8 μm-diameter superparamagnetic bead, for example, we found $z_{eq}$ = 0.714 μm. Such an equilibrium position however scales with the bead diameter *d*, as shown in Figure 3. Such a result is equivalent to the one obtained for ordinary Gaussian beam optical tweezers, in which we also have a constant $z_{eq}$/*d* ratio [26].

**Figure 4:**
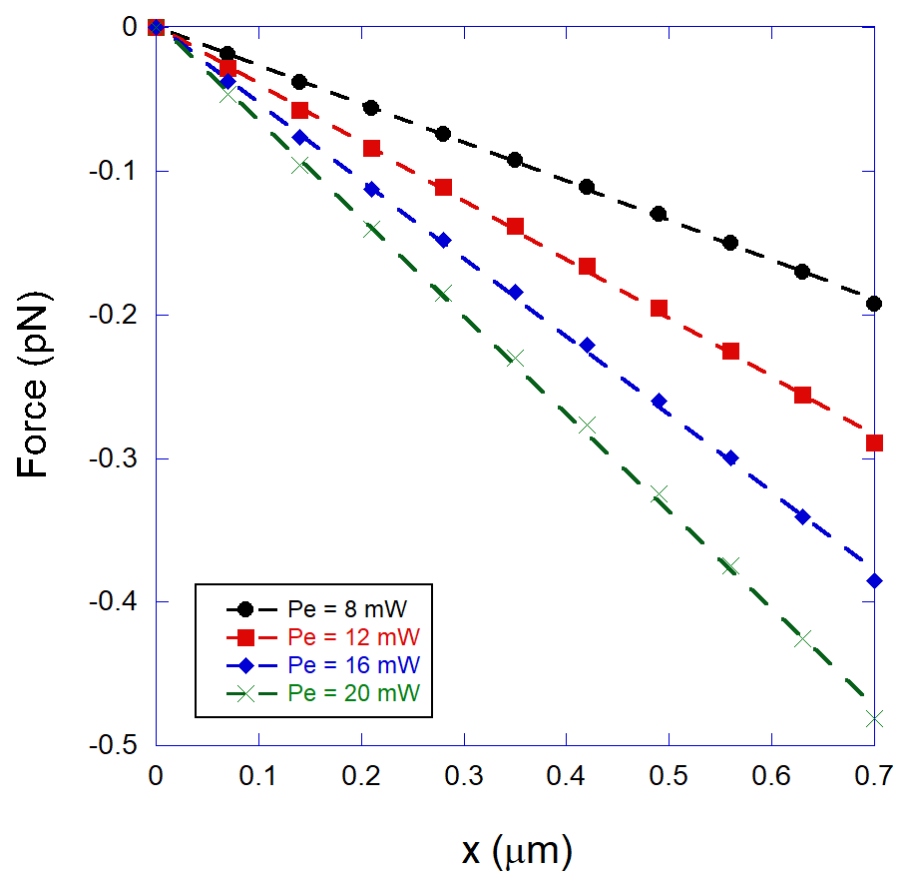
Calculated transverse optical forces as a function of the distance between the bead center and the equilibrium position, *x*, for a 2.8 μm-diameter bead, obtained for various different laser powers ($P_{e}$ denotes the power at the objective entrance).

Figure 4 shows some calculated optical forces plotted as a function of the distance between the bead center and the equilibrium position, *x*, for a 2.8 μm-diameter bead, obtained for various different laser powers ($P_{e}$ denotes the power at the objective entrance). Observe that the force increases linearly with the bead position, and the trap stiffness κ = −∂F/∂x increases with the laser power. Such results are again qualitatively equivalent to those obtained for ordinary Gaussian beam optical tweezers [26].

**Figure 5:**
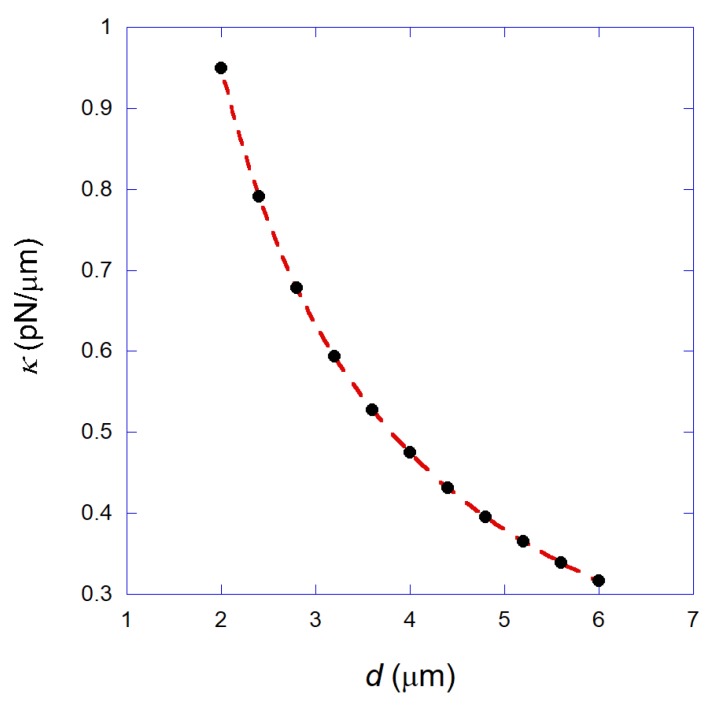
Black circles: Calculated transverse trap stiffness as a function of the bead diameter for the annular beam with $P_{e}$ = 20 mW. Dashed line: A fitting to a simple hyperbola.

Finally, Figure 5 shows the calculated transverse trap stiffness as a function of the bead diameter (black circles), obtained for a fixed laser power $P_{e}$ = 20 mW. The dashed line is a fitting to a simple hyperbola. This result shows that the trap stiffness of the annular beam tweezers presents a hyperbolic decay with the bead size, in a similar way as the ordinary Gaussian beam tweezers [26].

The numerical results presented above allow one to conclude that the annular beam tweezers work qualitatively equally to the ordinary Gaussian beam tweezers. An important difference is that the values obtained for the trap stiffness are considerably smaller than the equivalent results obtained for the original Gaussian beam tweezers using the same laser power at the objective entrance ($P_{e}$). This is due to the fact that the objective transmittance for the annular beam is much smaller, as discussed earlier. Nevertheless, as we discuss in the Appendix, if one normalizes the trap stiffness by the actual local power that reaches the trapped bead, we conclude that our annular-beam tweezers are equivalent to the original Gaussian beam tweezers in efficiency, presenting similar values of the trap stiffness normalized by the local laser power on the bead.

### 3.4. Experimental Results and Comparison with the Geometrical Optics Calculations

**Figure 6:**
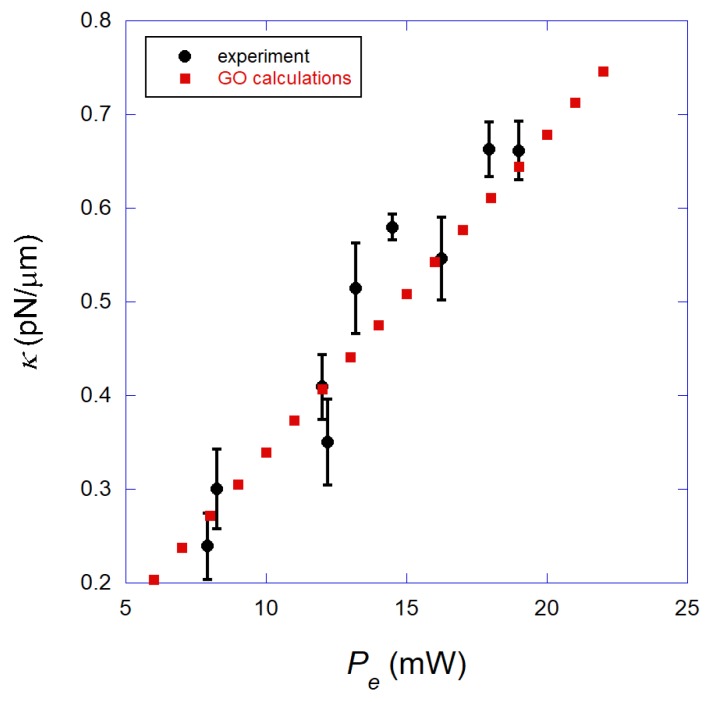
Black circles: Measured transverse trap stiffness κ of the annular beam optical tweezers as a function of the laser power $P_{e}$, measured at the objective entrance, obtained for a 2.8 μm-diameter superparamagnetic bead. Observe that κ increases linearly with the laser power, a result similar to that obtained for ordinary tweezers. Red squares: Theoretical prediction from our GO calculations. Observe that the agreement with the experimental data is excellent.

In Figure 6, we show the measured transverse trap stiffness κ (black circles) of the annular beam optical tweezers obtained for a 2.8 μm-diameter superparamagnetic bead, as a function of the laser power $P_{e}$, measured at the objective entrance. In all these measurements, the bead center was maintained at a height *h* = (4.4 ± 0.5) μm above the coverslip surface. Observe that the trap stiffness increases linearly with the laser power, a result similar to that obtained for ordinary tweezers that use Gaussian laser beams and dielectric beads. We also show the theoretical prediction of our GO calculations (red squares) obtained as described in the last section. Observe that the agreement between the experimental data and the theoretical prediction is excellent. This agreement justifies the use of the GO approximation for these beads. It also justifies the use of the number 1.6 for the refractive index of the superparamagnetic beads in the theoretical calculations, i.e., the iron content of the beads is not very important for the optical forces in this situation. Finally, the excellent agreement achieved between the experiments and theory indicates that light absorption by the superparamagnetic beads is small under our experimental conditions, using the annular laser beam with the parameters presented in Section 3.1. In fact, Iyengar et al. have previously demonstrated that the trap stiffness decreases with the laser power when there is a considerable absorption of light by the superparamagnetic particles, due to heating effects [14]. This is an intuitive result, since heating in fact tends to generate radiometric forces on the bead, which tends to push the particle away from the focal region, resulting in a more unstable trap [15,22].

It is worth emphasizing that the comparison performed between experiments and theory in Figure 6 is absolute, i.e., there were no adjustable parameters used in the theoretical calculations. In fact, all the parameters needed to obtain the calculated forces and trap stiffnesses were measured as described in Section 3.1 and Section 3.2.

**Figure 7:**
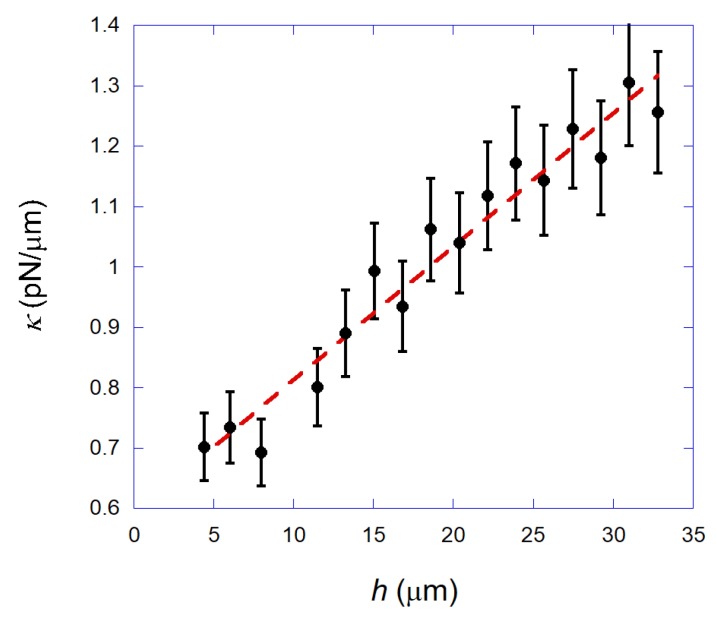
Black circles: Measured transverse trap stiffness κ as a function of the bead height *h* for a fixed laser power $P_{e}$ = 20 mW. Observe that the trap stiffness increases as a function of the bead height, a result opposite to that usually found for ordinary Gaussian tweezers. Dashed red line: A simple linear fitting only for guiding the eye.

In Figure 7, we show the transverse trap stiffness κ as a function of the bead height *h* (distance from the bead center to the coverslip surface) for a fixed laser power $P_{e}$ = 20 mW (measured at the objective entrance). Observe that the trap stiffness increases as a function of the bead height. Such a result is surprising, since in ordinary optical tweezers (Gaussian laser beam and dielectric beads), the trap stiffness usually decreases as a function of the bead height due to spherical aberration that occurs in the glass-water interface of the sample chamber. Such aberration degrades the laser focus and reduces the trapping efficiency [26,29].

Thus, our annular beam tweezers working with superparamagnetic beads present a major difference from ordinary optical tweezers: we have a system that works better far from the coverslip, i.e., tweezers that become stiffer for higher bead distances from the coverslip, at least for the height range studied here (<35 μm). Such behavior is due to the use of the annular beam in our system. In fact, a similar behavior was found when using such a beam for trapping ordinary dielectric polystyrene beads of 3 μm in diameter (see Appendix A). Unfortunately, such a behavior could not be captured by our simple geometrical optics calculations, suggesting that other effects must be considered.

## 4. Conclusions

In this paper, we propose a new optical tweezers setup based on an annular-shaped beam that is efficient at trapping superparamagnetic beads that cannot be easily trapped by conventional Gaussian beam tweezers. Such a property allows one to apply simultaneously optical and magnetic forces on these beads. In other words, it allows the implementation of a hybrid instrument that can use the best features of both optical and magnetic tweezers.

We have fully characterized the optical trapping of our superparamagnetic beads, comparing the experimental data with the theoretical predictions of a geometrical optics model. The results obtained and the conclusions drawn here provide new insights into the optical trapping and manipulation of superparamagnetic particles, which are important for the design of new future hybrid tweezers instruments.

## Figures and Tables

**Figure A1:**
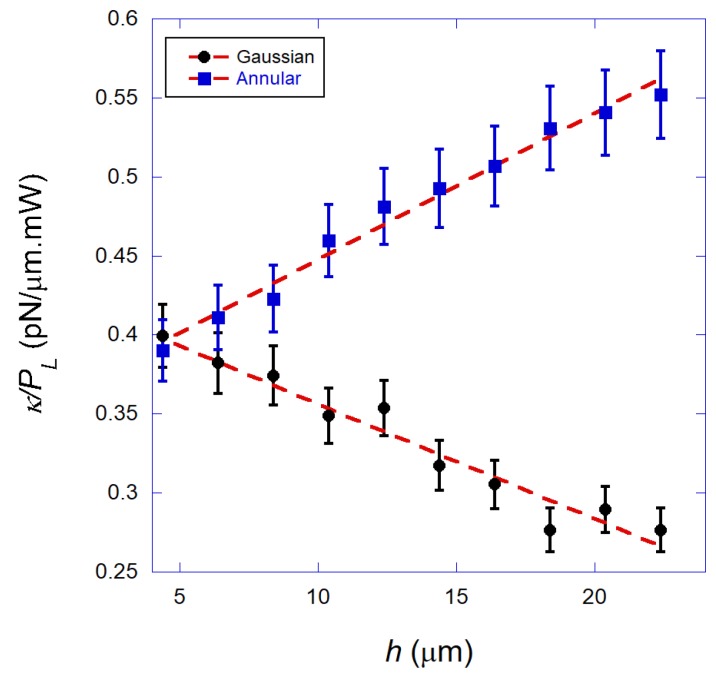
Blue squares: Transverse trap stiffness κ, normalized by the local power $P_{L}$ in the sample, as a function of the bead height *h* obtained for the annular beam tweezers using the 3-μm polystyrene beads. Black circles: Equivalent result obtained for the original Gaussian beam tweezers using the same 3-μm polystyrene beads. Observe that the results are qualitatively different, indicating that the beam geometry strongly interferes with the spherical aberration to which the trap is subjected. Dashed red lines: Simple linear fittings only for guiding the eye.

**Figure A2:**
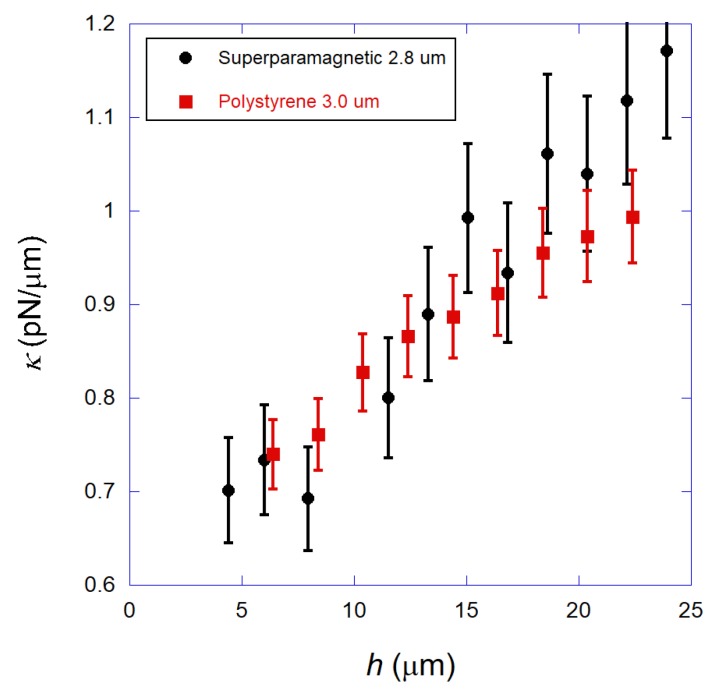
Comparison between the trap stiffness results obtained for the 3.0-μm polystyrene beads (red squares) and the for the 2.8-μm superparamagnetic beads (black circles). Despite the size difference between the two types of beads, the trap stiffness values are very similar within the error bars for the height range studied.

## Appendix A

Here, we advance the characterization of the annular-beam tweezers, showing a direct comparison between the results obtained for dielectric polystyrene beads of 3 μm in diameter (BangsLabs, Fishers, IN, USA) using both our annular beam and the original Gaussian beam tweezers. Note that such a comparison could not be performed using our superparamagnetic beads, since they cannot be stably trapped with our Gaussian beam tweezers.

The difference between the original Gaussian and the annular beam tweezers is the presence of the phase mask in the optical path, as depicted in Figure 1. Since this mask changes the actual laser power that reaches the objective entrance, here we will present measurements of the trap stiffness normalized by the laser power. In addition, since the objective transmittance is different for the two beam types, we normalize the trap stiffness by the local power $P_{L}$ in the sample, i.e., the actual laser power that reaches the trapped bead. The local power $P_{L}$ is obtained simply by multiplying the power measured at the objective entrance $P_{e}$ by the transmittance of the objective lens, which was determined as $T_{annular}$ = (9 ± 1)% for the annular beam and $T_{Gaussian}$ = (31 ± 1)% for the Gaussian beam (see Section 3.2).

In Figure A1 (blue squares), we show the transverse trap stiffness κ, normalized by the local power $P_{L}$ in the sample, as a function of the bead height *h* obtained for the annular beam tweezers using the 3-μm polystyrene beads. The equivalent result obtained for the original Gaussian beam tweezers using the same 3-μm polystyrene beads is also shown (black circles).

Observe that, for the annular beam tweezers, κ increases as a function of *h*, a behavior qualitatively identical to that found for the superparamagnetic beads under the same conditions. For the Gaussian beam tweezers, on the other hand, κ decreases as a function of *h*, a behavior well known for this type of optical tweezers, which is related to the focus degradation caused by spherical aberration on the glass-water interface of the sample chamber, an effect that reduces the trapping efficiency [24,26].

The results of Figure A1 strongly suggest that the unusual increase of κ measured for the annular tweezers is due to the beam geometry, as mentioned before. We believe that such a property is mainly due to the fact that the Gaussian beam is much more subjected to spherical aberration effects, because the rays’ angulation interval beyond the objective is much larger: from zero to the critical angle of the glass-water interface. In the case of the annular beam, spherical aberration is much less important, because the rays’ angulation interval is limited by the mask aperture. Thus, in the case of our annular beam, the focal region is much less spread spatially, and the trapping efficiency is less degraded. The fact that the stiffnesses obtained for the two beam geometries are basically the same for small bead heights (<5 μm) corroborates this conclusion, since spherical aberration effects are minimized as the bead approaches the coverslip surface [26]. Another important point to stress here is the fact that the Fourier transform of an annular beam is a Bessel beam [30]. Thus, since our beam is annular in the far field, it is expected that the beam beyond the objective should be close to a Bessel-like beam with non-diffracting characteristics, minimizing spherical aberration.

Finally, in Figure A2, we show a comparison between the trap stiffness results obtained for the 3.0-μm polystyrene beads (red squares) and the for the 2.8-μm superparamagnetic beads (black circles). Despite the size difference between the two types of beads, the trap stiffness values are very similar within the error bars for the height range studied. Such a result strengthens the previous conclusion that the iron content of the superparamagnetic beads does not interfere much with the optical forces for the annular beam geometry. It explicitly shows that these superparamagnetic beads can be used as ordinary dielectric beads in optical tweezers experiments if the annular beam is employed, with the advantage that the former can also be manipulated by a magnetic field generated in the sample region, thus allowing the implementation of hybrid optomagnetic traps.
